# Determining Spatial Summation and Its Effect on Contrast Sensitivity across the Central 20 Degrees of Visual Field

**DOI:** 10.1371/journal.pone.0158263

**Published:** 2016-07-06

**Authors:** Agnes Yiu Jeung Choi, Lisa Nivison-Smith, Sieu K Khuu, Michael Kalloniatis

**Affiliations:** 1 Centre for Eye Health, The University of New South Wales, Kensington, New South Wales, Australia; 2 School of Optometry and Vision Science, The University of New South Wales, Kensington, New South Wales, Australia; Dalhousie University, CANADA

## Abstract

**Purpose:**

Recent studies propose that the use of target stimuli within or close to complete spatial summation reveal larger threshold elevation in ocular disease. The Humphrey Visual Field Analyzer (HFA) is used to assess visual function yet the spatial summation characteristics are unexplored for the central macular region. We therefore wanted to establish the relationship between contrast sensitivity and stimulus size (spatial summation) within the central 20° visual field using the high sampling density of the 10–2 test grid.

**Methods:**

Thresholds were measured for one eye from 37 normal subjects using the HFA 10–2 test grid with all five Goldmann (G) targets (GI to GV). Subject data were converted to 50-year-old equivalent using published and calculated location-specific decade correction factors. Spatial summation curves were fitted for all data at all locations. The size of Ricco’s critical area (Ac) within which complete spatial summation operates (*k* = 1), and the slope of partial summation (*k* < 1: to characterize partial summation), was established.

**Results:**

The 50-year-old age normative data were determined for all Goldmann stimulus sizes for the 10–2 HFA test grid and showed a marked change in contrast sensitivity for small test stimuli (e.g. GI) and little change in larger test stimuli (e.g. GV). Both the Ac and *k* values did not vary with age allowing for the application of the age correction factors. Ac and *k* values increased with eccentricity with GI remaining within complete spatial summation and GII was close or within complete spatial summation. GIII or larger test sizes were always outside complete spatial summation operating within various levels of partial summation.

**Conclusions:**

The developed normative data now allows comparisons of data sets with high sampling density using the 10–2 grid irrespective of subject age. Test size is important when assessing ocular disease yet only GI or GII stimuli operate close to or within complete spatial summation in the macula. Current visual field testing protocols employ GIII which is always outside complete spatial summation and operates under various values of partial summation: GIII may not be the most suitable test size to assess ocular disease affecting the macula.

## Introduction

Static automated perimetry is a well-established test of visual function used routinely for the diagnosis and management of diseases affecting the visual pathway. White-on-white standard automated perimetry (SAP) is the most commonly performed clinical perimetry test protocol. [1–3] Contrast sensitivity is measured in SAP through the use of light increments at various locations in the visual field (VF), typically of ~100–200 ms duration. [1, 2, 4–6] Although five stimulus sizes (Goldmann size I to V) are available for testing in most commercial perimeters, the Goldmann size III (GIII) is the standard stimulus size used in conventional white-on-white perimetry. [4, 5, 7]

Contrast sensitivity depends upon the size of the test stimulus, in other words, it is governed by spatial summation. [8] This can be described by Ricco’s law [9] where luminance of a stimulus (*L*) x stimulus area (*A*) is a constant up to a critical area (*L* x *A*^*k*^ = *C*: with *k* = 1). That is, *L* and *A* are inversely proportional and the slope of an *L* vs. *A* graph plotted in log units is -1 when Ricco’s Law applies. Once the critical area (Ac) or Ricco’s area is reached, threshold operates under partial summation (0 < *k* < 1; also known as probability summation [10]) or no summation (when *k* = 0). Theories to explain this change include alterations in the size of the centre mechanism of centre/surround receptive fields of retinal ganglion cells or the extent of receptive fields of cortical cells. [11–16]

Goldmann [17] empirically determined what he referred to as the ‘summation exponent’, *k*, using kinetic perimetry comparing different sized targets and concluded that a *k* value close to 0.8 provided suitable isopter colocalization in the macular region. He also showed the need to increase target size with eccentricity to maintain a summation exponent close to unity. [17] Subsequent studies using both kinetic or static stimuli have confirmed Goldmann’s original observations and show that spatial summation is not uniform across the VF. [16, 18–20] For example, Khuu and Kalloniatis [21] characterised this change using the Humphrey Visual Field Analyzer (HFA) 30–2 test grid and found the Ac for many test points within the 30–2 test grid was smaller than that of the standard GIII used in routine clinical practice. These results suggest that conventional testing using a GIII target measures visual function within partial summation for the central ~40–50° diameter VF. [21]

**Fig 1:**
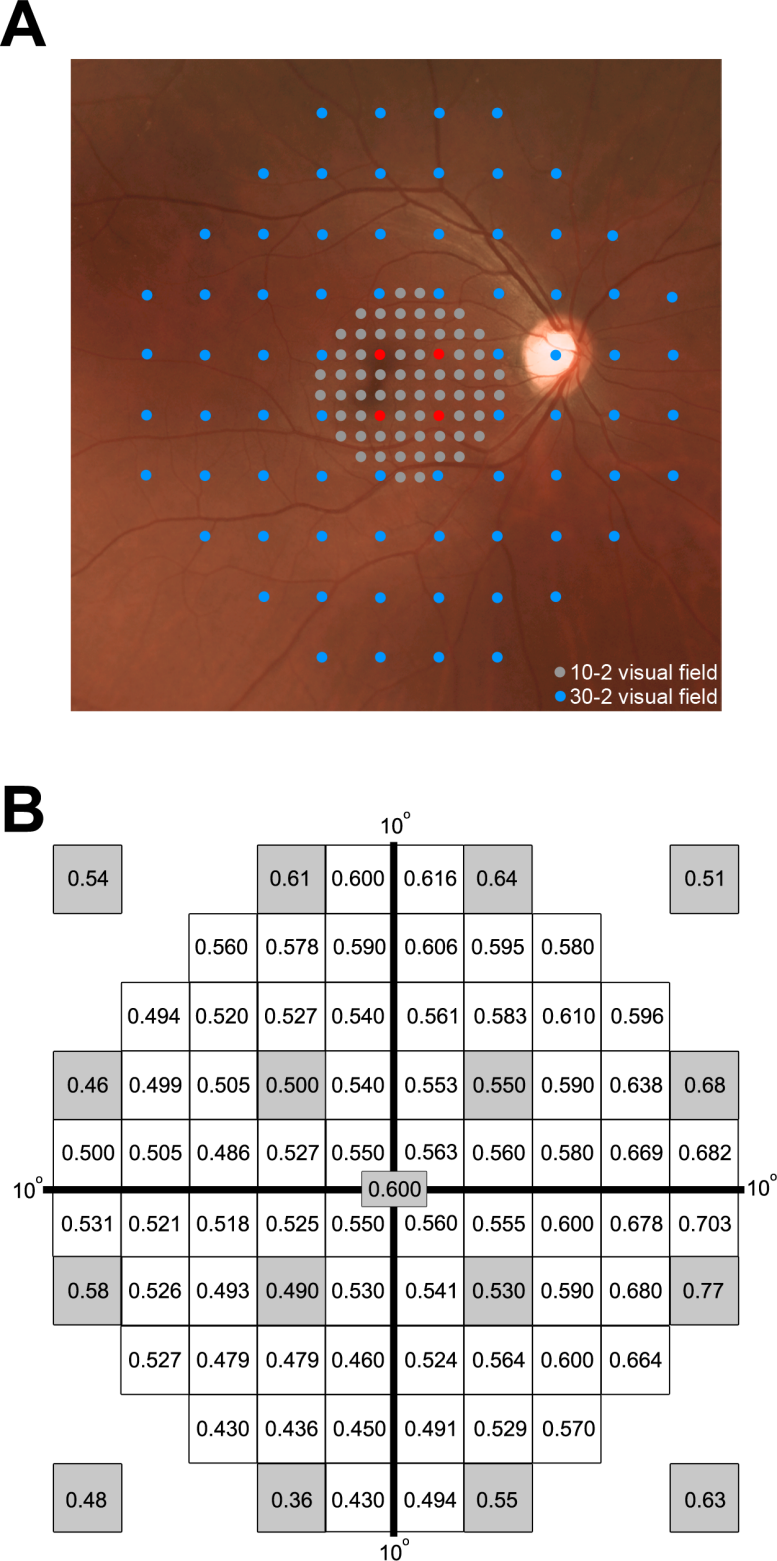
**Composite figure of visual field patterns (A) and estimated dB loss/decade (B).** (A) Fundus photograph with the 10–2 test pattern being superimposed on the 30–2 pattern showing the higher sampling within the central 20° diameter in the 10–2 pattern compared to the 30–2 test grid. The grey dots and blue dots belong to the 10–2 and 30–2 pattern, respectively. The red dots are common test points to the 10–2 and 30–2 test grids. (B) Estimated sensitivity loss (dB) per decade of life at each HFA 10–2 test location. Grey shaded locations indicate data extracted from Heijl et al. [5] All other values were calculated as a weighted average of the nearest three known correction factors from Heijl et al [5] and used to correct subject data to a single 50-year-old age equivalent.

In addition to the need to understand spatial summation within the macular region, visual sensitivity from the fovea to approximately 10° changes by ~0.8–0.9 log units for a GI test target and ~0.5 log units for a GIII [21] yet only four points sample at an eccentricity of 3° due to the 6° sampling strategy of the 30–2 grid. Conversely, the 2° sampling strategy of the 10–2 grid has many more points within the central 8–10° (Fig 1A). While spatial summation has been explored in the 30–2 test grid that extends to a diameter of 60°, [21] it is yet to be reported for the central VF using high density sampling. Specifically, the transition point from complete to partial spatial summation with different stimulus sizes in the central VF is unknown. This has clinical implications as the 10–2 test grid is highly relevant when central VF assessment is required in conditions such as glaucoma, [22–26] macular disease including age-related macular degeneration (AMD) [27–29] and other macular conditions. [30, 31]

Spatial summation is important when assessing ocular disease. Redmond et al [32] showed that the largest difference in visual function between glaucoma and age-matched normal subjects was observed when the stimulus size used was within complete summation while a smaller difference between the two was found when the stimulus sizes operates within partial summation. [32] Kalloniatis and Khuu [33] also demonstrated similar effects when using stimulus sizes that operate within complete summation using the 30–2 test grid of the HFA in a group of subjects with optic nerve disease. Hence, it is important to consider spatial summation in perimetry as it has implications for disease detection and monitoring as assessing visual function within complete versus partial summation may impact on the amount of contrast sensitivity loss detected in VF testing [4, 19, 20, 32, 33].

The current study explores spatial summation characteristics specific to the 10–2 high density test grid. We determined the spatial summation characteristics for all test locations within the central 10° of visual space and identified appropriate stimulus sizes for each test location such that thresholds are measured at or close to complete spatial summation. We also investigated the effect of age on Ac and slope of partial summation (*k*) in the spatial summation function to determine the suitability of age conversion for sensitivity data and the application of the principle equating contrast sensitivity for test stimuli operating within complete spatial summation using the dB* metric that includes test size in threshold reporting [21].

## Methods

### Subjects

Thirty-seven subjects with no history of visual abnormalities were recruited for the study. Subjects underwent a standard eye examination at the Centre for Eye Health (CFEH) at the University of New South Wales, including retinal photography (Kowa Non-Mydriatic Nonmyd WX3D) and optical coherence tomography (Macular Cube 512 A-scans x128 horizontal scan lines and Optic Disc Cube 200 A-scans x 200 horizontal scan lines; Cirrus OCT, Carl Zeiss Meditec) to confirm no detectable ocular pathology that would affect VF results. Table 1 outlines the characteristics of the subject cohort, including age distribution (20–62 years: mean 36±11 years and median 32 years) and visual acuities. Refractive errors ranged from +2.50 to -6D and astigmatic correction of <2.5D. Ethics approval was given by the University of New South Wales ethics committee in accordance with the tenets of the Declaration of Helsinki. All subjects gave written informed consent prior to the study.

**Table 1:** Characteristics of subject cohort for this study.

**Demographics**	
No of subjects	37
Male	14
Female	23
Mean age ± SD (years)	36 ± 11
Age range (years)	20–62
**Ocular parameters**	
Median best corrected visual acuity (BCVA)	20/16
BCVA range	20/25–20/16
Median refractive error (Rx)	-0.31
Rx range (spherical equivalent in diopters)	+2.50 to -6.00
**Ethnicity**	
Caucasian	15 (40.5%)
Asian	16 (43.2%)
Indian	3 (8.1%)
Caucasian/Hispanic	1 (2.7%)
Caucasian/Asian	2 (5.4%)

### Visual field testing

Threshold measurements (in dB) were obtained using the 10–2 HFA grid in Full Threshold mode (background luminance: 10 cd/m^2^; stimulus duration: 200 ms; stimulus size: GI to GV subtends 0.1°, 0.21°, 0.43°, 0.86°, and 1.72°, respectively). [6] Each subject was tested twice for each GI to GV and the final threshold measurement for each test location was averaged. All tests were performed monocularly (non-testing eye patched), with natural pupils and a refractive correction placed in the HFA trial frame if required. The short term fluctuation option was enabled to allow more than one threshold measurement to be obtained at some locations in a single test. The order of test size was randomised to minimise any order effects. Subjects were given breaks between tests to avoid fatigue. Tests were repeated if the reliability criteria were below those specified by the manufacturer or if the test-retest variability (as measured by the mean range of the threshold measurements at all locations) was greater than ~3dB. For analysis, data for each subject was converted to a right eye orientation.

### Definition of eccentricity of test locations

Test locations in the 10–2 test grid are 2° apart and arranged in a grid-like distribution (Fig 1A). The eccentricity of each location was calculated based on this as the distance from the centre of the test location to the foveal location in degrees. For the horizontal and vertical meridians, measurements for each eccentricity are based on the mean of two test locations on each side of the x-axis or y-axis respectively.

### Age correction of threshold measurements

**Fig 2:**
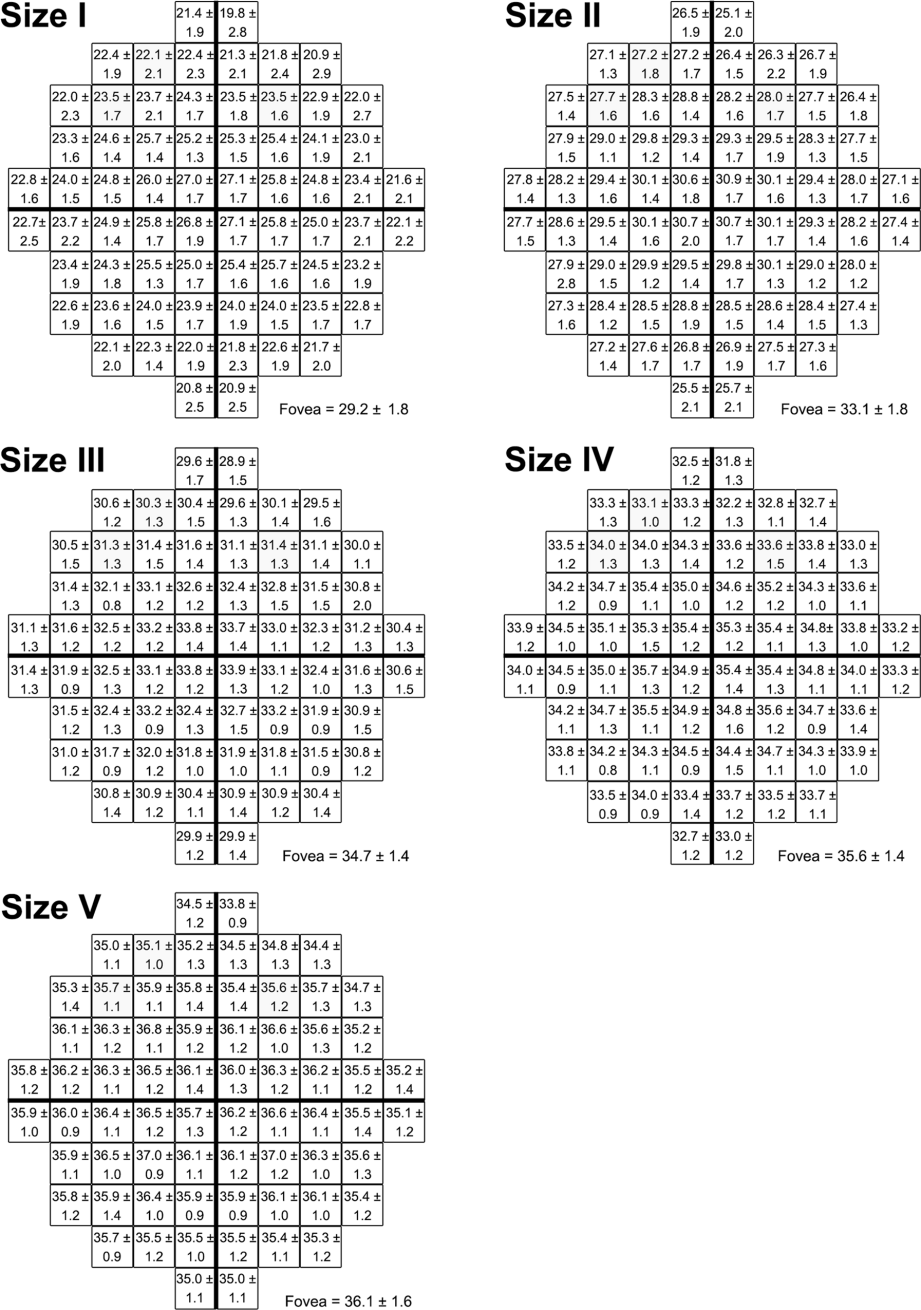
Mean contrast sensitivity (expressed as dB ± SD) at each HFA 10–2 test location. Mean contrast sensitivity values for all test locations with the five different stimulus sizes following correction of subject data to a 50-year-old age equivalent. These values can be converted to be equivalent to any other age by utilising Fig 1B.

Subject data was corrected to a single age equivalent as previously reported [21] following confirmation that spatial summation parameters were age independent for all participants (see S1 Text). Data were corrected to a 50-year old equivalent as study participants were of different ages and therefore it was inappropriate to average raw, non-age corrected dB values considering that contrast sensitivity is known to reduce with age. [5, 34] Correction factors were determined based on the rate of age-induced sensitivity loss determined in Heijl et al [5] for the 30–2 test grid. Thirteen points including the foveal point were directly translated from Heijl et al [5] to their equivalent locations in the 10–2 test grid (Fig 1B). Correction factors for the remaining locations of the 10–2 test grid were then calculated as the average of the nearest three known values from Heijl et al [5]. Known values were weighted before averaging according to their distance to the location of interest. For some locations, factors were averaged from four known values as two values were equidistant from the location of interest. Correction values were then used to adjust the threshold measurements of each participant to 50 year-old equivalent dB values. For example, the foveal threshold value for a GI stimulus for a 30-year old participant is derived from multiplying 0.6 (foveal age correction factor in Fig 1B) by the difference between 50 and 30 divided by 10 (i.e. (50–30)/10) = 2) then subtracting this product from 29.2 (50-year old equivalent foveal threshold for GI in Fig 2) (i.e. 29.2 –(2 x 0.6) = 28 dB).

### Establishing Ac and slope of partial summation

Age-corrected threshold measurements were converted to units of dB*, established by Khuu and Kalloniatis. [21] The dB* equates sensitivity value for test size differences and assign a uniform sensitivity value irrespective of test size if within Ricco’s law. The dB* value is derived from the sum of dB and a size factor (in dB). [21] Based on the 0.6 log unit difference for each Goldmann test size area, a 6 dB size factor was associated for each test size. [21] Thus when contrast sensitivity is expressed in dB*, a GI stimulus, for example, would effectively give the same contrast sensitivity value as a GII stimulus if both stimuli are operating within complete spatial summation. This is useful as one could predict the contrast sensitivity value given by a stimulus size that shares the same spatial summation characteristics as another stimulus size that has a known contrast sensitivity value at a particular test location (see Kalloniatis and Khuu 2016 [33] for detailed discussion).

To determine spatial summation functions, threshold measurements (in dB*) for each test location were plotted as a function of log stimulus size area and fitted with a two-line, segmental linear regression with a least squares approach in GraphPad Prism (v6, GraphPad Software, Inc., La Jolla, CA, USA). The first line was constricted to a gradient of zero and a second line allowed to vary. The Ac value was taken as the inflection point of the bilinear function. The slope of partial summation (*k*) was estimated using the limited stimulus sizes available on the HFA (Wilson [16] has shown a curvilinear relationship when a large range of stimulus sizes were available). We report the slope of partial summation as a negative value (i.e. *k* was calculated as if the data were plotted in a log L versus log A plot).

### Statistical analysis and curve fitting routine

Statistical analysis was performed using GraphPad Prism. Contrast sensitivity (in dB and dB*) and Ac and slope of partial summation (*k*) values were analysed using a two-way ANOVA with repeated measures. A two-way ANOVA analysis was performed to determine how stimulus size and VF eccentricity affect contrast sensitivity (in units of dB and dB*), Ac and slope of partial summation (*k*). We hypothesized that contrast sensitivity, Ac and *k* are all affected by stimulus size and VF eccentricity. Standard deviation (SD) of sensitivity (dB) values were analysed using an ordinary two-way ANOVA. Tukey’s multiple comparison tests (corrected for multiple comparisons at an α of 0.05) were conducted to compare dB, dB*, and SD values at each eccentricity between different test sizes. Linear regression analysis was used to compare subject age with Ac values and partial summation slope values for each test location (see S1 Fig). We assumed no significant (α = 0.05) interaction if the slope of the fitted line did not deviate from zero. Normal distribution of dB values at each test location was confirmed using the Kolmogorov-Smirnov normality test (with Dallal-Wilkinson-Lillie for an α = 0.01) in GraphPad Prism.

## Results

### Normative threshold measurements across the 10–2 test grid

**Fig 3:**
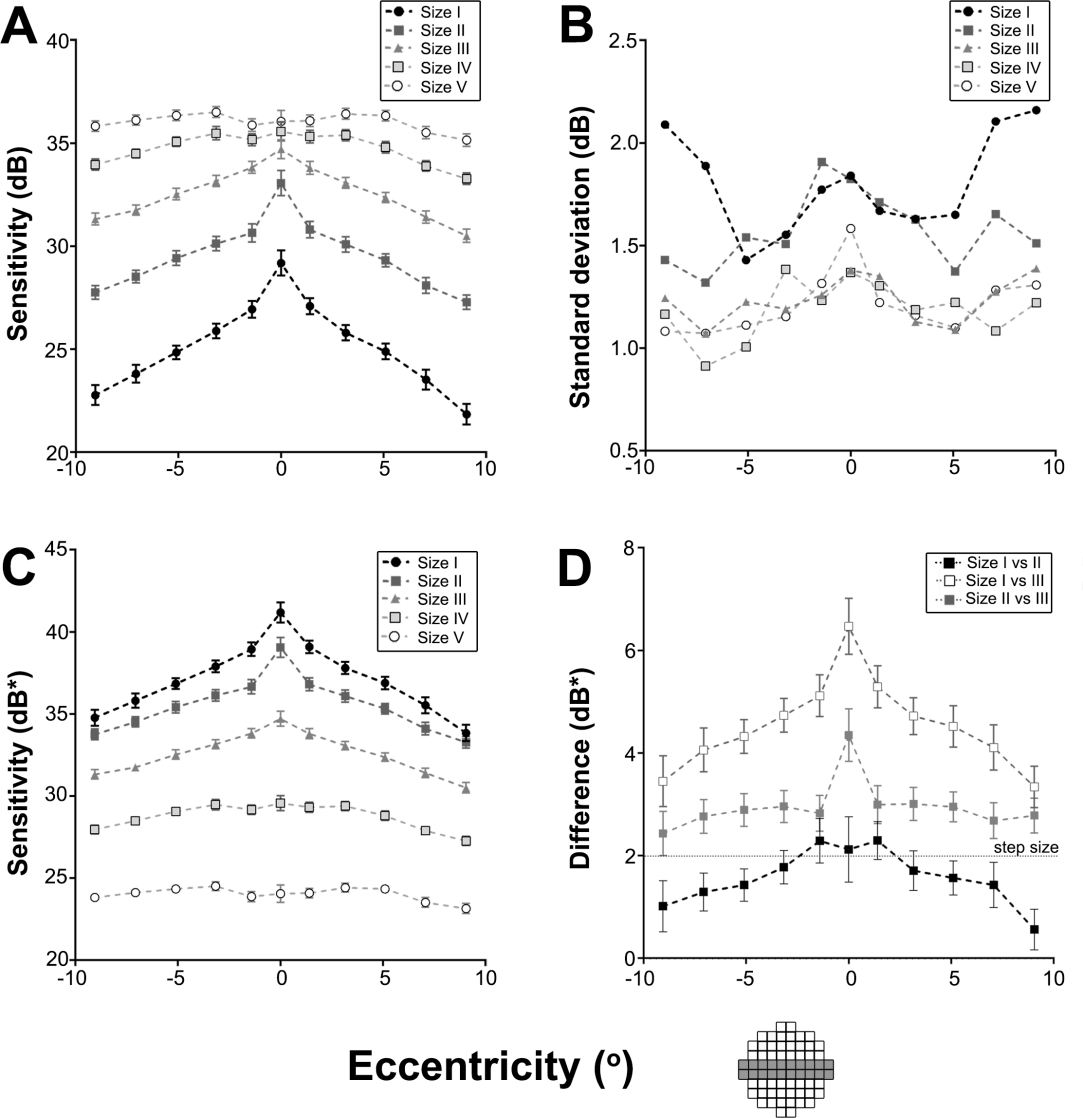
Composite figure showing contrast sensitivity characteristics along the horizontal meridian in the 10–2 test grid. (A) Contrast sensitivity values along the horizontal meridian (shaded in the 10–2 test grid diagram) with the five stimulus sizes were plotted to demonstrate the Hill of Vision. (B) SD of contrast sensitivity values were plotted to show variability in sensitivity with eccentricity and stimulus size. (C) Data along the horizontal meridian (shaded in the 10–2 test grid diagram) with the five stimulus sizes were converted to equated contrast sensitivity (dB*) and plotted. (D) The difference in equated contrast sensitivity (dB*) along the horizontal meridian (shaded in the 10–2 test grid diagram) between GI and GII, GI and GIII, and GII and GIII were plotted. This demonstrates that GII was nearer Ricco’s area than GIII given that GI was within Ricco’s area. The dashed line represents the maximum error in threshold estimation of 2dB in the Full Threshold Algorithm. All data points represent the mean and error bars represent 95% confidence intervals.

The 50-year old equivalent average threshold measurements at each test location in the 10–2 test grid for GI to GV are shown in Fig 2. For all stimulus sizes, sensitivity values decreased with increasing VF eccentricity. This effect was best visualised in sensitivity profiles generated from age-corrected dB values along the horizontal meridian (Fig 3A). Sensitivity profiles also highlighted the increase in sensitivity values with increasing stimulus size. These effects were significant based on a two-way ANOVA that showed a main effect for eccentricity (*F*[10, 50] = 738.3, *P* < 0.0001) and stimulus size (*F*[4, 5] = 15294, *P* < 0.0001) along the horizontal meridian. The analyses also showed significant interaction effects indicating that change in contrast sensitivity with eccentricity is significantly dependent on stimulus size (*F*[40, 50] = 64.62, *P* < 0.0001). For example, the mean decrease in sensitivity from the fovea to the temporal edge of the 10–2 test grid was 7.3 dB for GI but only 0.9 dB for GV across the same eccentricity range (Fig 3A).

We then investigated the variability in threshold measurements between stimulus sizes to determine if this played a role in the differences in sensitivity profiles for each stimulus size along the horizontal meridian. We found overlapping standard deviation (SD) profiles for GIII, IV and V (Fig 3B) and similar SD values for GI to GII except in more peripheral locations. An ordinary two-way ANOVA showed eccentricity (*F*[10, 40] = 3.141, *P* = 0.0047) and stimulus size (*F*[4, 40] = 39.34, *P* < 0.0001) had a significant effect on SD but post-hoc tests showed SD values were not significantly different between GI and the conventional GIII within 5.1° from the fovea. SD values at locations beyond 5.1° were significantly different between GI and GIII; for example, at an eccentricity of 9.1° temporally (95% CI: 0.1900–1.352). However, differences were not clinically significant as they were lower than the intra-individual variation (i.e. < 3 dB on average). [5]

### Sensitivity (dB*) as a function of eccentricity

Fig 3C is a transformation of Fig 3A with age-corrected threshold values in dB from Fig 3A converted to threshold values in dB* to equate stimulus size [21]. Similar to the sensitivity (dB) profiles, a two-way ANOVA reported a main effect of both stimulus size (*F*[4, 5] = 22561, *P* < 0.0001) and eccentricity (*F*[10, 50] = 738.3, *P* < 0.0001) and significant interaction effects indicating equated contrast sensitivity was dependent on both eccentricity and stimulus size (*F*[40, 50] = 64.62, *P* < 0.0001). Tukey’s multiple comparison tests (corrected for multiple comparisons at an α of 0.05) were conducted to compare the mean dB* values at each eccentricity along the horizontal meridian between different stimulus sizes. Post hoc tests showed that dB* values for GI and GII were significantly different at the fovea (95% CI: 1.720–2.520) and at all eccentricities. The difference in dB* values between stimulus sizes (e.g. GI and GII, GI and GIII, and GII and GIII) observed in Fig 3C was depicted by a difference plot in Fig 3D. The mean dB* difference along the horizontal meridian was 1.6 (between GI and GII), 4.6 (between GI and GIII), and 3.0 (between GII and GIII). The mean dB* difference between GI and GII was less than the maximum error in threshold estimation of 2dB given the smallest step size in the staircase is 2dB in the Full Threshold Algorithm. [35]

### Normative Ac values across the 10–2 test grid

**Fig 4:**
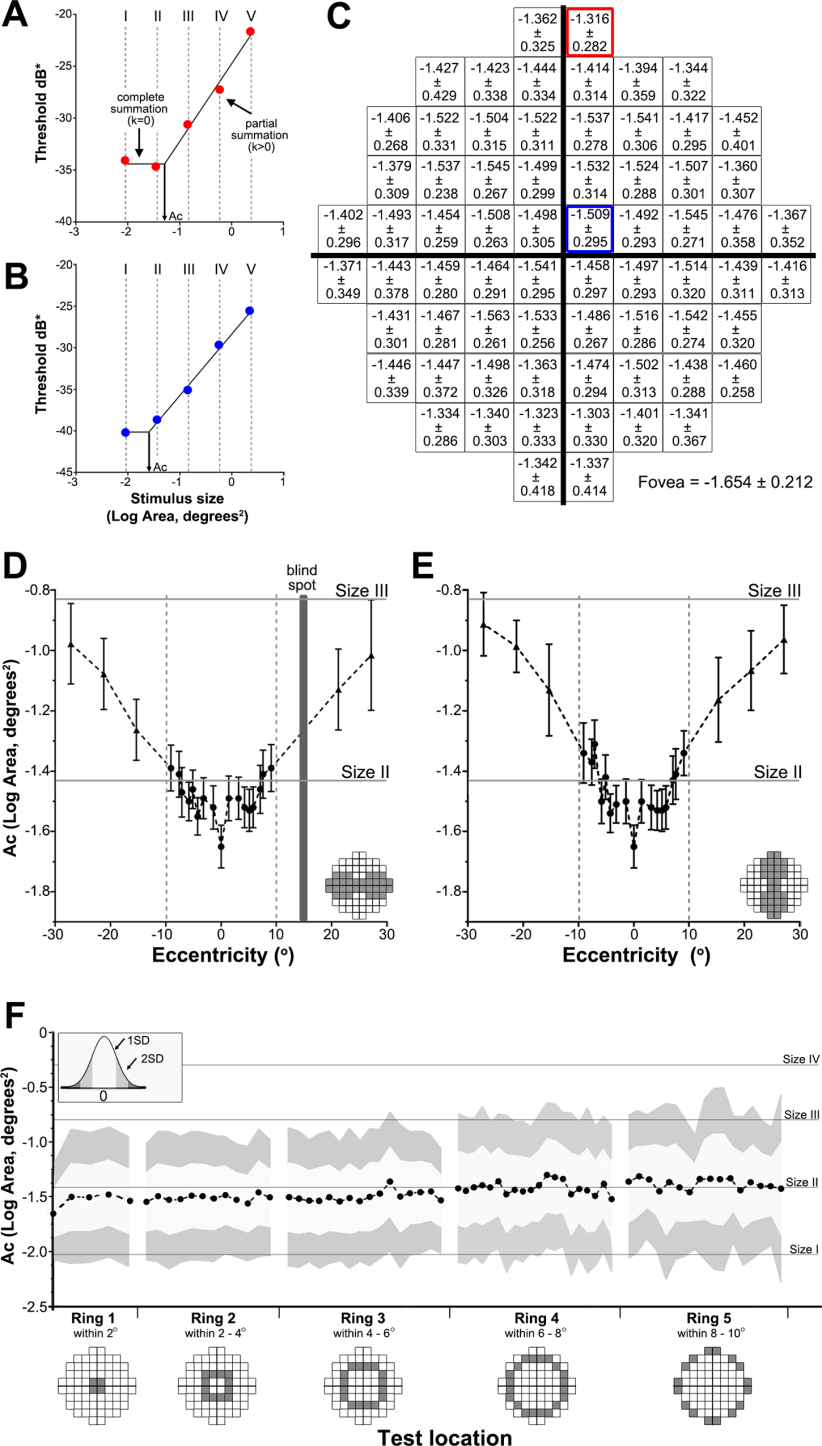
Ac characteristics in the 10–2 test grid. (A-B) Examples of spatial summation graphs for two test locations. Contrast sensitivity (expressed in dB* which is simply adding a size factor to the conventional dB with a factor of 0 dB for GIII, +12 dB for GI, +6 dB for GII, -6 dB for GIV and -12 dB for GV, based on the 0.6 log unit difference between the Goldmann stimulus sizes) was plotted against stimulus size (as Log Area, Degrees^2^) for test locations within (A) 2° of the fovea and (B) 6° of the fovea in the 10–2 test grid for a single subject following correction of subject contrast sensitivity data to a 50-year-old age equivalent and conversion to equate contrast sensitivity for test size and depicted by the dB* value. Ac was determined by the inflection point (arrow) of the bilinear function. (C) Ac values across all subjects (mean ± SD) for each test location in the 10–2 test grid. (D-E) Ac values (mean ± 95% confidence intervals) were plotted for the (D) horizontal and (E) vertical meridians (shaded in the 10–2 test grid diagrams). Values within the central 10° (inside the dotted lines) indicate data from the current study and data for locations beyond 10° (outside the dotted lines) indicate data extracted from Khuu and Kalloniatis 2015. [21] (F) Ac values were grouped into rings based on eccentricity and plotted as mean ± 1 SD (light grey shading) and 2 SD (dark grey shading). The foveal Ac is included in ring 1.

Spatial summation graphs were constructed for all test locations for all subjects individually and the Ac value at a test location determined from the inflection point (Fig 4A and 4B). The mean Ac value of all subjects at each test location across the 10–2 test grid is shown in Fig 4C. Along the horizontal meridian, change in Ac was not significant between nasal and temporal locations (*F*[1,2] = 0.9101, *P* = 0.4408; Fig 4D). Along the vertical meridian however, there was a significant difference between superior and inferior locations (*F*[1,2] = 649.8, *P* = 0.0015; Fig 4E). Ac values showed a significant increase with eccentricity for both the horizontal and vertical meridians (*F*[8,16] = 21.28, *P* < 0.0001; *F*[8,6] = 37.01, *P* < 0.0001, respectively). Ac values also increased with eccentricity when analysed concentrically within the 10–2 test grid; mean Ac of test locations within 2˚ of the fovea (Fig 4F, ring 1) was -1.532 (range: -1.654 to -1.458) compared to a mean Ac value of -1.383 (range: -1.460 to -1.316) for test locations 8–10˚ away from the fovea (Fig 4F, ring 5). The mean Ac values for test locations within 6° of the fovea (Fig 4F, rings 1, 2, 3) were all smaller than the standard GIII stimulus (log area, degrees^2^ = -0.831) and GII stimulus (log area, degrees^2^ = -1.433) suggesting only a GI stimulus (log area, degrees^2^ = -2.035) would lead to a threshold response within complete spatial summation. For the majority of test locations that were between 6˚ to 10˚ from the fovea (Fig 4F, rings 4 and 5) the mean Ac was -1.419 and -1.383 respectively indicating the largest possible stimulus size for complete spatial summation would be a GII stimulus (log area, degrees^2^ = -1.433).

**Fig 5:**
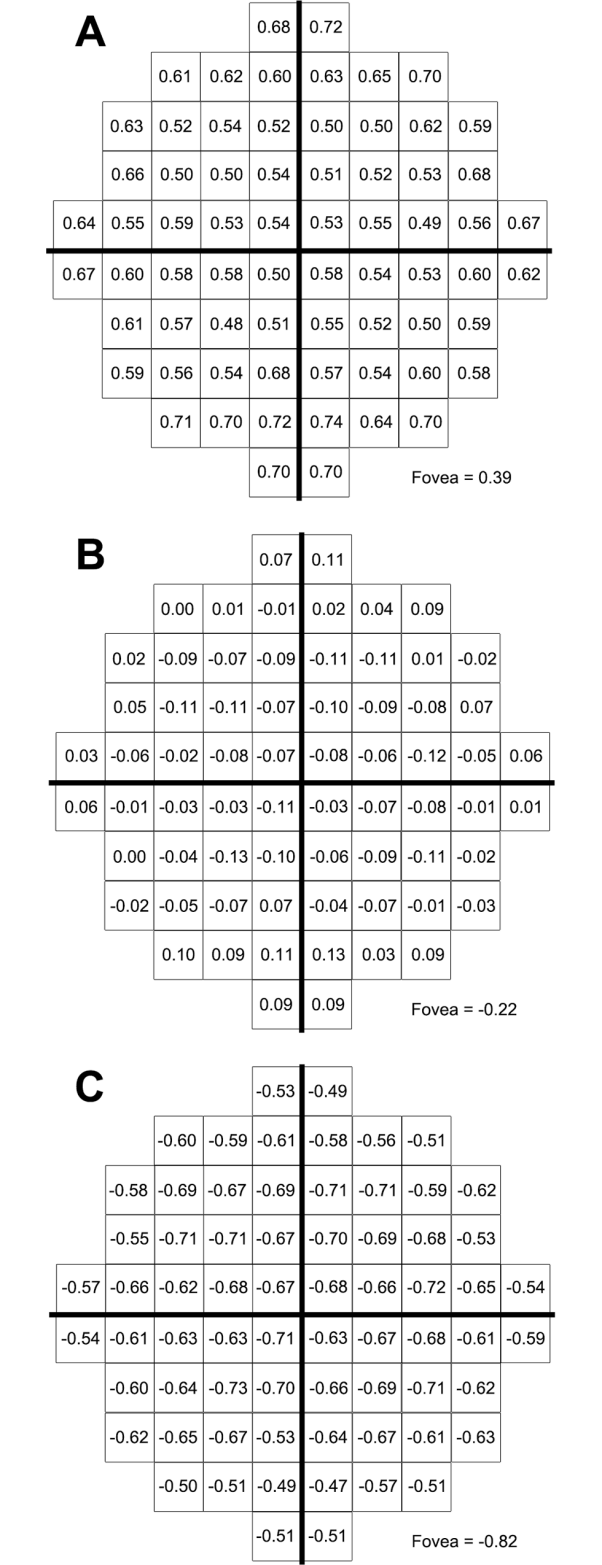
**Resultant value when the Goldmann target size I (A) size II (B) and size III (C) is subtracted from the critical area at each of the 10–2 locations.** A negative value of -0.20 or less indicates locations outside a summation exponent (*k*) of 0.8. GII is the largest Goldmann test size that operates at a summation exponent of 0.8 or larger at all locations of the 10–2 grid except the fovea.

Fig 5 provides a difference plot of the critical area derived from the data shown in Fig 4C subtracting the area of the different Goldmann test sizes (GI, GII and GIII in panels 5A, 5B and 5C). A positive value indicates that the stimulus is always within complete spatial summation (Fig 5A for GI), while a value of -0.2 or higher indicates that the test size is within the summation exponent value of *k ≥* 0.8 (Fig 5B for GII). GIII was always at least 0.47 log units outside complete spatial summation (values were always negative) and thus within partial summation at all test locations of the 10–2 grid (Fig 5C). We therefore wanted to determine the degree of spatial summation GIII and larger test sizes operate within the macular region.

### Partial summation slopes across the 10–2 test grid

**Fig 6:**
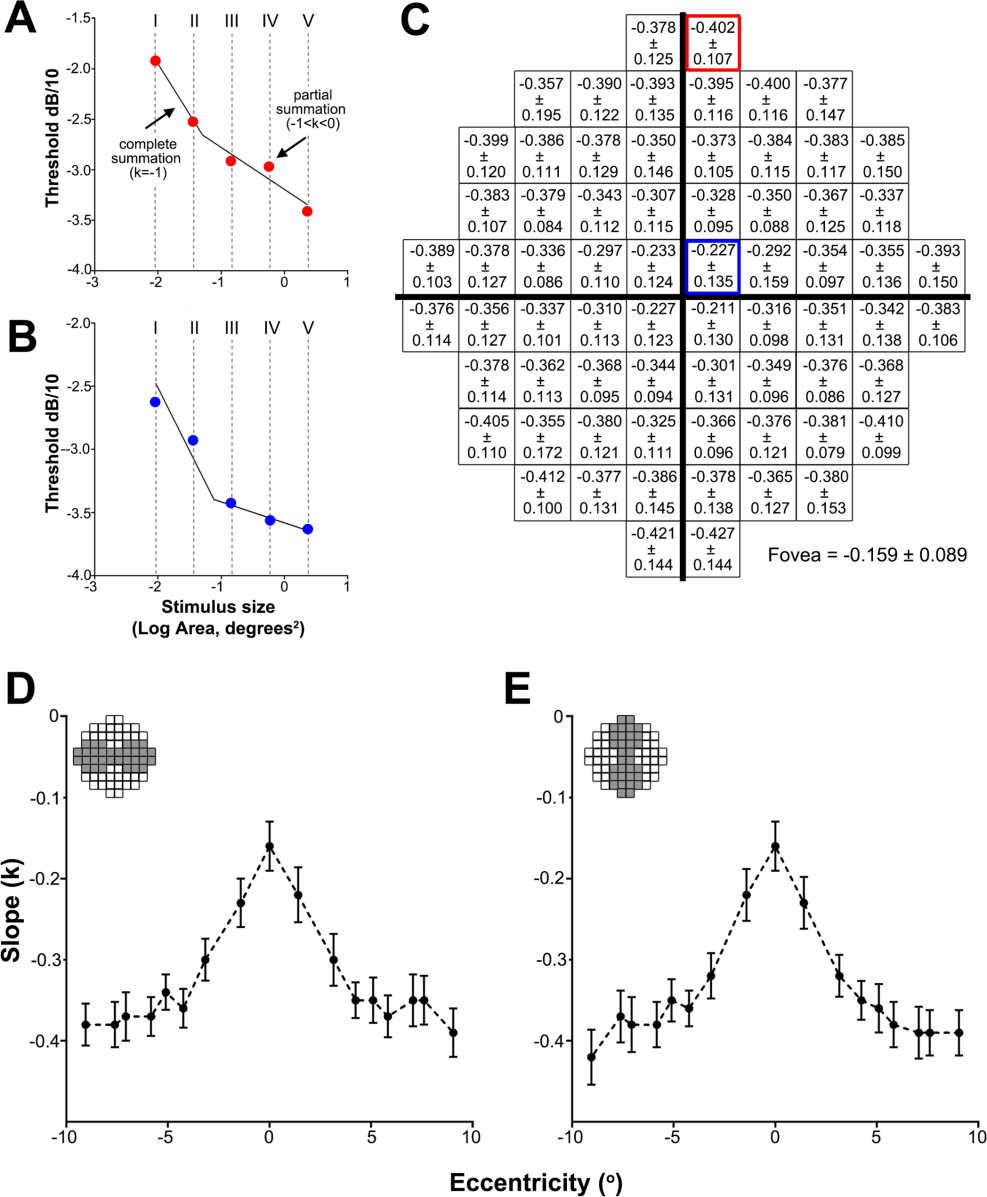
Slope of partial summation for the 10–2 test grid. The slope of partial summation (i.e. *k*) on spatial summation graphs with contrast sensitivity (as dB/10) being plotted against stimulus size (as Log Area, Degrees^2^) for test locations within (A) 2° of the fovea and (B) 6° of the fovea for a single subject. (C) Partial summation (*k*) values averaged across all subjects (mean ± SD) for each test location in the 10–2 test grid. Mean k value ± 95% confidence intervals were plotted for the horizontal (D) and vertical (E) meridians.

The mean slope values (*k*) across the 10–2 test grid and the horizontal and vertical meridians are shown in Fig 6. Along the horizontal meridian, change in *k* was not statistically significant between nasal and temporal locations (*F*[1,1162] = 0.6647, *P* = 0.4151; Fig 6D). Similarly, there was no significant difference between superior and inferior locations along the vertical meridian (*F*[1,1157] = 0.02464, *P* = 0.8753; Fig 6E). However, *k* values showed a significant increase (i.e. steepening slope) with eccentricity for both the horizontal and vertical meridians (*F*[8,1162] = 56.48, *P* < 0.0001; *F*[8,1157] = 60.90, *P* < 0.0001, respectively). For example, mean *k* at the fovea was -0.159±0.089 compared to -0.393±0.150 at the temporal edge of the 10–2 test grid (Fig 6D).

## Discussion

To our knowledge, this is the first study to investigate spatial summation using the 10–2 grid that tests at 2° intervals in the central 20° of the VF. This current paper provides new knowledge of the contrast sensitivity change and changes in spatial summation within a region of rapid sensitivity change that is poorly sampled by the 30–2 test grid. While Khuu and Kalloniatis [21] have established that the GIII stimulus is larger than Ricco’s area for most points in the 30–2 test grid, details of spatial summation in the central VF was not possible in this study due to the coarse sampling density of the 30–2 test grid. We have established that GI and GII are near or within complete spatial summation within the central 20° of the visual field, and importantly we established the transition between the two Goldmann stimuli which occurs at ~6° from the fovea. That is, only GI operates within complete spatial summation within the 6° of the fovea while GII operates within complete spatial summation for most test locations that were between 6° to 10° from the fovea.

We measured contrast sensitivity and characterised spatial summation characteristics including establishing normative Ac and partial summation slope (*k*) values for all test locations within the 10–2 grid. We observed an increase in Ac with VF eccentricity in all quadrants, similar to previous studies [16, 19, 21, 32] and a steepening slope of partial summation with eccentricity. In addition, we found that mean Ac values for all locations within the 10–2 test grid were smaller than the GIII stimulus currently used in standard clinical protocols suggesting normal testing paradigms are not operating within the critical area but within various levels of partial summation across the 10–2 test grid.

There are two key issues that emerge with the understanding of spatial summation within the 10–2 grid outlined in this study. The first relates to the potential usefulness of the 10–2 high density sampling and whether the high density is beneficial for various test sizes. The steepness of the contrast sensitivity profiles for GI and GII targets suggests that it should be possible to derive isocontrast profiles that are of higher density to those obtained for test stimuli with flatter slopes in the contrast sensitivity profile, e.g., GIII and larger. Our preliminary work using clustering algorithms followed by statistical testing using the methods outlined by Kalloniatis et al [36] appears to support this proposition. The sampling density for GI and GII appears to reflect suitable sampling strategy for the 10–2 grid, while GIII and above is oversampling reflected by a lower number of isocontrast contours (larger isocontrast areas). The second issue relates to the use of smaller stimulus sizes at or within complete spatial summation resulting in increased threshold variability. The ‘signal-to-noise ratio’ changes with stimulus size and eccentricity (see Fig 3B), with greater variability observed with a GI particularly at peripheral locations. This observation is entirely consistent with the work of Wall et al [37] who also noted an increase in variability (flatter psychometric function) when smaller stimulus sizes are employed in visual field testing. To maximise sampling density while minimising variability, our data suggests that GII may be appropriate for testing using the 10–2 paradigm as it operates near if not within complete spatial summation and that it shows less variability compared to GI. Future work in identifying threshold elevation in ocular disease will determine the suitability of GII to identify larger thresholds weighed against a poorer signal-to-noise ratio.

### Normative Ac values for the 10–2 test grid are consistent with previous data

Our Ac values at the fovea were comparable to those observed in Khuu and Kalloniatis [21] for the 30–2 test grid (-1.745 ± 0.225 in Khuu and Kalloniatis [21] vs. -1.654 ± 0.212 in our study) and at the four other spatially equivalent central locations (e.g. -1.576 ± 0.225 in the point adjacent and superonasal to the fovea in Khuu and Kalloniatis [21] vs. -1.545 ± 0.267 in our study). Our Ac values at two locations at 10° eccentricity superiorly (-1.362 ± 0.325 and -1.316 ± 0.282) were also comparable to those reported by Redmond et al [32] for a similar test location (-1.29 ± 0.51). Importantly, our Ac values demonstrated an increase with eccentricity, similar to previous studies [16, 18, 19, 21].

### Usefulness of the sensitivity (dB*) profile

Our results showed that the GI stimulus always operates within complete spatial summation in the 10–2 test grid. If dB* values of different stimulus sizes overlap in the sensitivity profile (Fig 3C), this reflects that the test stimuli operate within complete spatial summation at that eccentricity. We found that the difference in dB* values between GI and GII is the least compared to that between GI and the other sizes (i.e. GIII-GV) suggesting that GII operates nearer complete spatial summation compared to the other sizes (i.e. GIII-GV). Our results and previous studies [19, 21, 38] show that GI always operates within complete spatial summation in the central VF (Fig 5A). Therefore the sensitivity (dB*) profile (Fig 3C) as well as the difference plot (Fig 3D) may serve as a surrogate for how near a stimulus size is operating within complete spatial summation. Further, the difference plots shown in Fig 5 now allow the use of GII rather than GI, [33] for testing when using the central four points if the 10–2 test grid is used to maintain stimulus size within or close to complete spatial summation.

### Standard Goldmann size III stimulus tests outside complete spatial summation in the 10–2 grid and operates within partial summation

The spatial summation characteristics explored in this study have implications for current clinical testing paradigms as visual function quantified using GIII would not reflect a direct, one-to-one inverse relationship between threshold luminance and stimulus area as defined by Ricco’s law. [9] The difference plots shown in Fig 5 show only GI and GII are within or close to complete spatial summation for the 10–2 test grid. The standard GIII routinely used in this paradigm operates under various levels of partial summation that change with eccentricity, starting relatively flat at the fovea then rapidly becoming steeper until at about 5° eccentricity, after which little variability occurs (Fig 6). The reason for this abrupt change in the partial summation slope at about 5° eccentricity is not known. Interestingly, a somewhat similar profile is seen in ganglion cell density where there is a rapid decline in the density of ganglion cells away from the fovea. [39, 40] Whether there is any association between the two observations would require further research. Partial or probability summation is thought to occur when the outputs of independent detectors are combined on the basis of probability, such that as more detectors are stimulated, the probability of detecting the stimulus also increases due to a greater probability that at least one detector will catch the stimulus. [41–43] Considering that GIII operates within partial summation, the change in the slope of partial summation (*k*) with eccentricity observed in our study suggests that the detection of GIII may be mediated by different underlying mechanisms across the 10–2 test grid. However, the role of partial summation in the presence of ocular disease when some of these detection mechanisms may drop out is unknown.

### Applications of spatial summation in the 10–2 test grid in disease and structure-function concordance

The findings reported here for the 10–2 test grid have highly relevant applications to clinical assessment of disease. Routine VF testing often employs the 30–2 or 24–2 test grids. [44] However, major eye diseases such as AMD and glaucoma report visual defects within the central 5° and superior paracentral 3° respectively. [27, 45] Detection of central vision loss is likely to be poor in the 30–2 or 24–2 test grids as the macular region is only sampled by 4 points. [45, 46] However, the 10–2 test grid has a high central sampling strategy and has shown significant efficacy in the assessment of glaucoma [22–26] and macular disease. [27, 29, 30] The normative contrast sensitivity values given in this study serve as a useful reference for clinicians to integrate the 10–2 test grid into routine clinical assessment of disease. In addition, the Ac values described here allow spatial summation to be considered when using the 10–2 test grid for disease detection. Test size is important when assessing threshold elevation in ocular disease: test targets close to or within complete spatial summation display higher threshold [32] and more anomalous test points and poorer global indices (mean deviation and pattern standard deviation) in a group of patients with optic nerve disease [33].

There have been a number of studies as reviewed by Malik et al [47] that attempted to compare structural and functional data. The ‘structure-function dissociation’ issue was raised in their review paper that large clinical trials have shown that structural changes may precede or occur after functional changes, and that only a small percentage of patients showed concurrent structural and functional changes. [47–50] More recently, Raza and Hood [51] evaluated several models and found an upper *R*^*2*^ values (the coefficient of determination) of 0.64. These results suggest that the structure-function relationship is not well predicted from these models. We propose that measuring VF thresholds using stimuli within or close to complete spatial summation may be one way to strengthen the ‘structure-function association’.

### Limitations of the study

Limitations include the size of the sample and the use of the 50-year-old equivalent to allow comparisons across the ages. The fitting paradigm only uses the five Goldmann test sizes and as such, may be prone to higher error as well as the inherent change in the number of points available for the fitting of the two-line spatial summation functions with changing eccentricity.

## Conclusion

We have established normative threshold sensitivity for GI to GV, Ac values and the slope of partial summation across the 10–2 test grid that operates within the macular region. This normative data provides the foundations for future studies to compare contrast sensitivity, Ac and *k* values in ocular disease. Our Ac values indicate that the standard GIII stimulus operates within various levels of partial summation across the macular region and never within complete spatial summation. Future studies using GI or GII test targets will test the hypothesis that a greater loss is found within ocular disease when testing within or close to complete spatial summation.

## Supporting Information

S1 Fig Ac and *k* as a function of age. Comparison of subject age with (A) Ac and (B) *k* showing neither value is dependent on age. Values indicate the slope of the linear regression fitted to the data for each test location in the 10–2 test grid which were all not significantly different from 0 except locations denoted by *. Representative graphs and regression lines are shown on the left for the two shaded test locations (locations 13 and 30) in the 10–2 test grid. Error bars represent 1 standard error of the mean.
(TIF)

S1 Text Age correction. (DOCX)
