# Supplementary figures and images for: Determining Spatial Summation and Its Effect on Contrast Sensitivity across the Central 20 Degrees of Visual Field
Source: PLoS One. 2016 Jul 6;11(7):e0158263. doi: 10.1371/journal.pone.0158263 (PMC4934771; doi:10.1371/journal.pone.0158263)

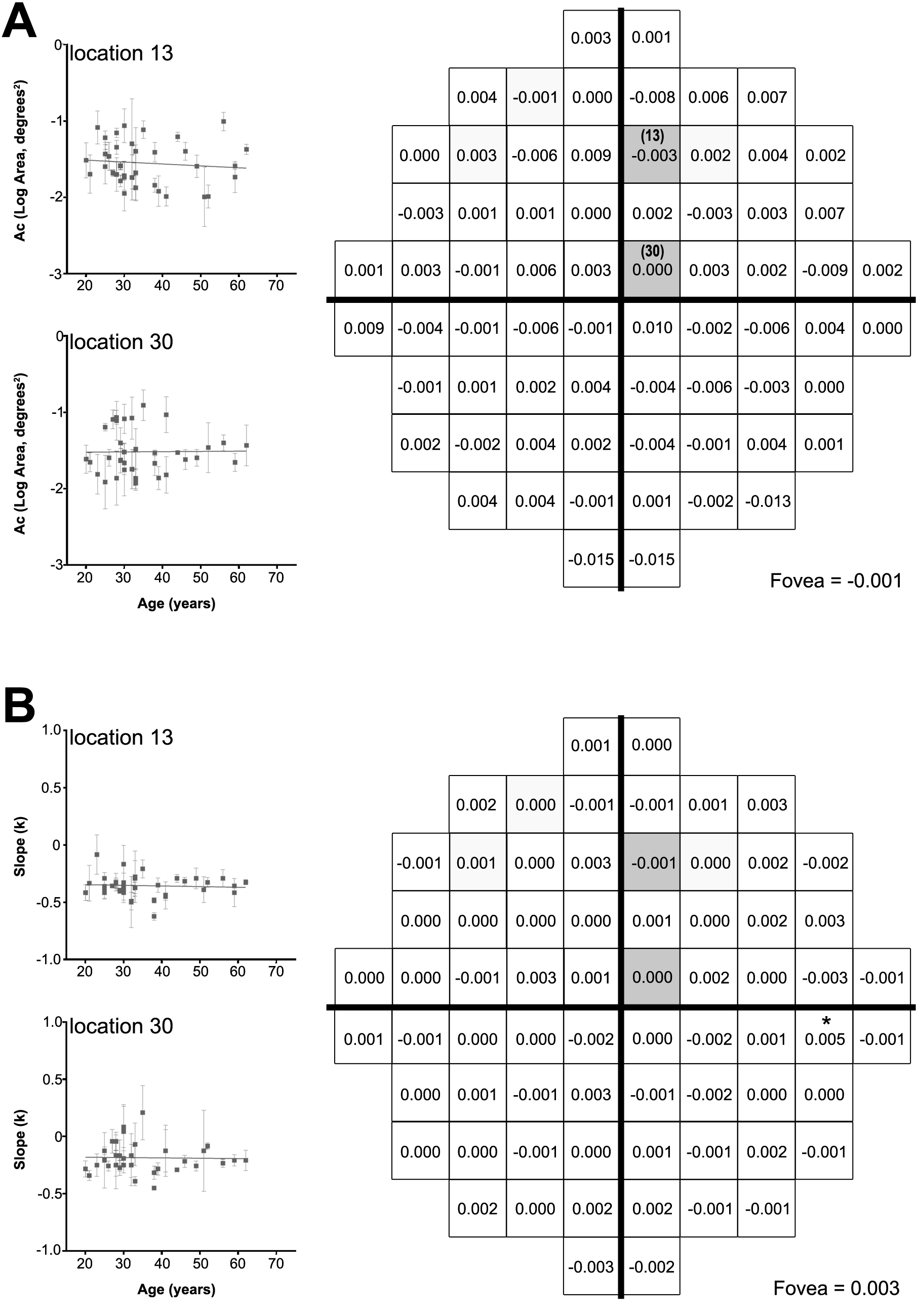

Supplement: S1 Fig — Comparison of subject age with (A) Ac and (B) k showing neither value is dependent on age. Values indicate the slope of the linear regression fitted to the data for each test location in the 10–2 test grid which were all not significantly different from 0 except locations denoted by *. Representative graphs and regression lines are shown on the left for the two shaded test locations (locations 13 and 30) in the 10–2 test grid. Error bars represent 1 standard error of the mean. (TIF) [file pone.0158263.s001.tif]
