# Supplementary material for: Determining Spatial Summation and Its Effect on Contrast Sensitivity across the Central 20 Degrees of Visual Field
Source: PLoS One. 2016 Jul 6;11(7):e0158263. doi: 10.1371/journal.pone.0158263 (PMC4934771; doi:10.1371/journal.pone.0158263)
Supplement: S1 Text — (DOCX) [file pone.0158263.s002.docx]

**Supporting Information**

**S1 Text. Age correction**

To establish that subject data could be corrected to a single age equivalent (as in Khuu and Kalloniatis [1]), Ac values for all subjects were confirmed to be age independent. Briefly, contrast sensitivity values (dB) for each subject at each test location was plotted as a function of log stimulus area and data fitted with a bilinear function to obtain a spatial summation plot. Ac and *k* values were taken as the inflection point and gradient of the second line of the bilinear function respectively. Values were plotted against age (S1 Fig) and linear regression analysis was performed to investigate the relationship between Ac, *k* and age. The slope of the line of best fit for Ac did not significantly deviate from zero for all test locations suggesting no correlation between Ac and age (e.g. at location 13, slope = -0.003, *P* = 0.559, *R^2^* = 0.010). The slope of the line of best fit for *k* did not significantly deviate from zero for all test locations except location 43 (slope = 0.005, *P* = 0.009, *R^2^* = 0.188). This suggests no correlation between age and *k*. These findings are consistent with previous studies. [1, 2]

**References**

1. Khuu SK, Kalloniatis M. Standard automated perimetry: determining spatial summation and its effect on contrast sensitivity across the visual field. Investigative ophthalmology & visual science. 2015;56:3565-76.

2. Redmond T, Zlatkova MB, Garway-Heath DF, Anderson RS. The effect of age on the area of complete spatial summation for chromatic and achromatic stimuli. Investigative ophthalmology & visual science. 2010;51(12):6533-9.
